# Hearing Loss in Offspring Exposed to Antiretrovirals During Pregnancy and Breastfeeding

**DOI:** 10.3389/fnmol.2022.920528

**Published:** 2022-06-20

**Authors:** J. Riley DeBacker, Breanna Langenek, Eric C. Bielefeld

**Affiliations:** ^1^VA RR&D National Center for Rehabilitative Auditory Research, VA Portland Healthcare System, Portland, OR, United States; ^2^Oregon Hearing Research Center, Oregon Health and Science University, Portland, OR, United States; ^3^Department of Speech and Hearing Science, The Ohio State University, Columbus, OH, United States

**Keywords:** antiretroviral therapy, ototoxicity, HIV, translational model, sensorineural hearing loss, auditory brainstem response

## Abstract

Over 27 million people worldwide currently receive daily antiretroviral therapy for the management of HIV/AIDS. In order to prevent the continued spread of HIV, the World Health Organization (WHO) recommends the use of highly active antiretroviral therapy by pregnant and nursing women. There is currently little research into the auditory effects of this therapy on children exposed during pregnancy and breastfeeding, and research to date on the direct effects of antiretroviral exposure on the auditory system is inconclusive. The current study examined the effects of WHO-recommended first-line antiretrovirals in a well-controlled animal model to evaluate the potential for auditory damage and dysfunction following these exposures. Female breeding mice were each exposed to one of four antiretroviral cocktails or a vehicle control once daily during pregnancy and breastfeeding. Offspring of these mice had their auditory status evaluated after weaning using auditory brainstem responses and distortion-product otoacoustic emissions (DPOAEs). Auditory brainstem response thresholds following antiretroviral exposure during gestation and breastfeeding showed elevated thresholds and increased wave latencies in offspring of exposed mice when compared to unexposed controls, but no corresponding decrease in DPOAE amplitude. These differences in threshold were small and so may explain the lack of identified hearing loss in antiretroviral-exposed children during hearing screenings at birth. Minimal degrees of hearing impairment in children have been correlated with decreased academic performance and impaired auditory processing, and so these findings, if also seen in human children, suggest significant implications for children exposed to antiretrovirals during development despite passing hearing screenings at birth.

## Introduction

Human immunodeficiency virus (HIV) is an acquired viral infection that suppresses the immune system and leaves those infected more prone to opportunistic and latent disease. The World Health Organization (WHO) estimates that, at the end of 2020, there were nearly 38 million people living with HIV and that more than 27 million of those individuals were taking lifelong antiretroviral (ARV) therapy for HIV management (WHO, 2020). Highly-active antiretroviral therapy (HAART), also commonly referred to as “combination antiretroviral therapy,” has been used since 1996 to manage symptoms of HIV and prevent disease progression and seroconversion to acquired immunodeficiency syndrome (AIDS). HAART typically consists of two nucleoside reverse transcriptase inhibitors (NRTIs) and one or more other drugs that have been shown to be effective not only at disease management but also at lowering disease burden to a point that prevents transmission (Cohen et al., 2011). As the WHO and other public health entities have a goal of preventing HIV transmission, HAART is recommended for all people living with HIV, and special focus has been paid to preventing the spread of HIV from mother to child during pregnancy (vertical transmission). While the use of HAART during pregnancy and breastfeeding has been shown to be effective in preventing the spread of HIV, exposure to HIV and HAART during development has been found to have negative impacts on cognitive development (Blanche et al., 1999; Tardieu et al., 2005; Brogly et al., 2007; Coelho et al., 2017; McHenry et al., 2019), language development (Rice et al., 2013), and auditory function (Poblano et al., 2004; Fasunla et al., 2014; Torre et al., 2017).

Current evidence regarding the auditory effects of HAART on perinatally HIV exposed but uninfected (PHEU) children has been inconclusive. Fasunla et al. (2014) found that *in utero* HIV exposure was more likely to result in failed hearing screening and confirmed hearing loss on a diagnostic auditory brainstem response (ABR), with a significant relationship between maternal viral load during pregnancy and hearing loss, but no relationship between CD4+ cell count and hearing loss. This study suggests that there may be some relationship between pre- and peri-natal HIV exposure and congenital hearing loss, but it did not control for whether or not mothers in the study were taking ARV therapy during pregnancy. Another study found significant delays in the Wave I latency and I-III interpeak latency on the ABR for PHEU infants exposed to the HAART drug zidovudine (AZT) alone or in combination with lamivudine (3TC; Poblano et al., 2004). In contrast, Torre and colleagues found that no specific HAART drug was related to an increased likelihood of hearing screening failure in PHEU children and that exposure to the drug tenofovir disoproxil fumarate (TDF) during the first trimester was associated with a lower odds ratio for a failed hearing screening. The authors also noted that a number of HAART drugs demonstrated an incredibly wide range of variability in auditory outcomes for newborns, even after controlling for factors that often contribute to failed hearing screenings (Torre et al., 2017). Despite the lack of differences in hearing screening results at birth, the Torre group also found that PHEU young adults were more likely to have impaired words-in-noise performance with otherwise normal cognition than young adults with HIV, suggesting an effect of exposure to these drugs during pregnancy and breastfeeding (PaB) not seen from post-natal exposures (Torre et al., 2020).

A prospective controlled human study of the relative contributions of HIV and HAART to hearing loss would be unethical, due to the high efficacy of HAART in preventing vertical HIV transmission, and so this question should first be explored in a well-controlled non-human model. Our group undertook an initial exploration of this modeling by exposing C57BL6/J female mice to AZT and 3TC during PaB (DeBacker et al., 2022). When offspring of these mice underwent ABR threshold testing at three weeks old, they had higher thresholds than control offspring at five of six tested frequencies. This indicates that exposure to AZT+3TC during PaB can lead to auditory dysfunction during development in a mouse model. AZT+3TC alone is not a currently recommended first-line management regimen for HIV, however, and so there is interest in understanding if these effects are seen across different currently-recommended ARV combinations. The current study seeks to expand upon our previous work by evaluating the auditory system of animals exposed to several different currently-recommended ARV combinations.

The current study used a well-characterized model of ototoxicity, the CBA/CaJ mouse, to investigate the auditory effects of HAART exposure during PaB. This model was used due to its stable hearing thresholds over the projected length of the study, in contrast to models with earlier onset of presbycusis like the C57BL6/J used previously. The authors hypothesized that exposure to HAART would lead to increased ABR and distortion product otoacoustic emissions (DPOAE) thresholds at wean in exposed offspring when compared to unexposed controls, with the greatest threshold elevation resulting from exposure to AZT and efavirenz (EFV). This hypothesis was based upon the previously-discussed findings on PHEU children and a study of the effects of ARV compounds on auditory cell (HEI-OC1) lines by Thein et al. (2014). This study found that exposure to moderate- and high-dose EFV resulted in almost 100% cell death and that even low-dose exposures cause significant cell death. While TDF was more toxic than AZT in the Thein et al. (2014) study, Torre et al. (2017) found a decrease in reported hearing screening failures following TDF exposure, and so it was predicted that AZT would have greater auditory effects than TDF. When combined with previous work by Thein et al. (2014) and our lab (DeBacker et al., 2022) on combination ARVs, it was anticipated that this study of WHO-recommended first-line HAART cocktails would result in greater auditory impairment than was observed in our previous study. By using currently-recommended HAART cocktails, this model provides a clinically translatable model of HAART exposure and contributes significant pre-clinical evidence toward the understanding of the auditory effects of HAART exposure during PaB.

## Methods

### Subjects

One hundred CBA/CaJ mice were used in this study. Of these mice, 20 were breeding mice obtained from Jackson Labs (Las Vegas, NV) and housed in a vivarium at The Ohio State University. The breeding mice were divided into breeding pairs and then assigned to one of five experimental groups. The other 80 mice were offspring of those pairs. Each experimental group consisted of four breeding mice and 10–16 offspring. In order to exclude confounding variables, the male breeder mice were not exposed to HAART or any other manipulation during the study. Breeding pairs were allowed to generate no more than five litters before removal from the study. The mice were kept in a quiet colony, in which the 24-h dB Leq level never exceeded 45 dB SPL. Animals were acclimatized to the colony for at least 7 days before beginning experiments. All procedures involving the animals were approved by The Ohio State University’s Institutional Animal Care and Use Committee.

### Antiretroviral Exposures

For all experimental arms and conditions, the following groups were used: one group’s (Group 1) breeder females received volume-matched distilled water vehicle; the other four groups’ (Groups 2–5) breeder females received 3TC combined with the following drugs: Group (2) TDF and EFV; Group (3) AZT and EFV; Group (4) TDF and nevirapine (NVP); Group (5) AZT and NVP. These drug cocktails correspond to permutations of HAART currently recommended by WHO for first-line therapy for pregnant and nursing women, though emtricitabine is also recommended as an alternative to 3TC, which was used in this study. The combinations were chosen because significant differences were seen in cellular toxicity between EFV and NVP and TDF and AZT, respectively, but no such differences were observed between emtricitabine and 3TC (Thein et al., 2014).

All drugs used in this study were obtained as capsules or tablets through The Ohio State University Wexner Medical Center Pharmacy. AZT and TDF tablets were crushed using a mortar and pestle, and distilled water was added to dissolve them and create stock solutions with a concentration of 10 mg/ml. 3TC tablets were crushed using a mortar and pestle, and distilled water was added to create a stock solution with a concentration of 50 mg/ml. Suspensions were made using the combinations of 3TC with AZT or TDF, depending on the experimental group. For groups receiving NVP, tablets were crushed and added to the suspension. For groups receiving EFV, capsules were emptied directly into the suspension. After adding EFV or NVP, 4 ml of water were added to each suspension, and they were thoroughly mixed to minimize particulate in each jar. All jars were agitated prior to administration to minimize particulate in the suspensions. Suspensions were refrigerated between administrations. Each day, suspensions were monitored for an irregularity in appearance prior to administration. After 28 days, any remaining suspension was discarded, and new suspensions were mixed.

Because the female breeder mice grew in size and weight over the course of the study, the doses of the HAART compounds increased as well. However, best practice standards set the maximum fluid volume that could be delivered to the mice through oral gavage at 0.20 ml. Therefore, in order to deliver the required doses without exceeding the maximum fluid volume, the concentrations in mg/ml of the compounds needed to increase, and so after 3 months, concentrations of the HAART suspensions were recalculated to reflect the higher weight of the animals at that time. After this recalculation, concentrations of AZT and TDF were 13 mg/ml, concentrations of EFV and NVP were 83 mg/ml, and concentration of 3TC was 68.2 mg/ml. Preparations were otherwise unchanged from the above procedure.

Each female breeding mouse was given a once-daily dose *via* oral gavage of one of the four cocktails of antiretroviral agents listed earlier in this section or a matched volume of vehicle solution for control subjects in Group 1. Daily doses were administered beginning after baseline testing and continued until the final group of offspring used for the study was weaned. As such, female mice were exposed during the mating period, pregnancy, and nursing of all offspring. Weights to determine dosing were collected on the first day of each week and were used for the duration of that week unless a mouse gave birth. After giving birth, the previous week’s weight was used for the remaining doses during that week. All gavage doses were delivered in a sterile environment under a biosafety hood in the University Laboratory Animal Resource housing vivarium.

### Auditory Brainstem Responses

For this study, all animals were anesthetized using an inhaled mixture of gaseous isoflurane (2.5% for induction, 1.2% for maintenance) and oxygen (2 L/min for induction, 1 L/min for maintenance) during both ABR and DPOAE collection. ABR and DPOAE testing was performed in a sound-attenuating booth (Controlled Acoustical Environments, Bronx, NY).

For eliciting the ABRs, tone bursts were presented beginning at 90 dB SPL and in decreasing 5 dB steps to 20 dB SPL or until no repeatable waveform was observed. Test frequencies were 4, 8, 12, 16, 24, and 32 kHz. The stimuli were generated using Tucker Davis Technologies (TDT, Gainesville, FL) SigGen software. Each tone burst was 1 ms in duration and had a 0.5 ms rise/fall time with no plateau. Stimuli were presented at a rate of 19/s. Signals were routed to a speaker (TDT Model MF1) positioned at 90 degrees azimuth (directly next to the right ear), 3 cm from the vertex of each mouse’s head. The levels were calibrated with a SoundTrack LxT1 sound level meter (Larson Davis, Depew, NY) with a $\frac{1}{4}$ in condenser microphone (model 7016 and model 4016, ACO Pacific, Inc.), placed at the level of the animal’s head. For recording electrical responses from the mice, three 6-mm platinum electrodes (Rochester Electro-Medical, Lutz, FL) were inserted subdermally behind the right pinna (inverting), behind the left pinna (non-inverting), and in the right rear leg (ground) of each anesthetized mouse. The evoked responses of the mice were amplified with a gain of 50,000× using a TDT RA4LI headstage connected to a TDT RA4PA preamplifier. ABRs were averaged across 300 responses at each level. Responses were processed through a 300–3,000 Hz band-pass filter as recommended by the software manufacturer (TDT). Post-acquisition analyses were performed using TDT BioSig RZ software. ABR P1 latencies were obtained by placing cursors at the positive P1 peak, and P3 latencies were obtained using the same process for the third positive peak.

### Distortion-Product Otoacoustic Emissions

**Figure 1:**
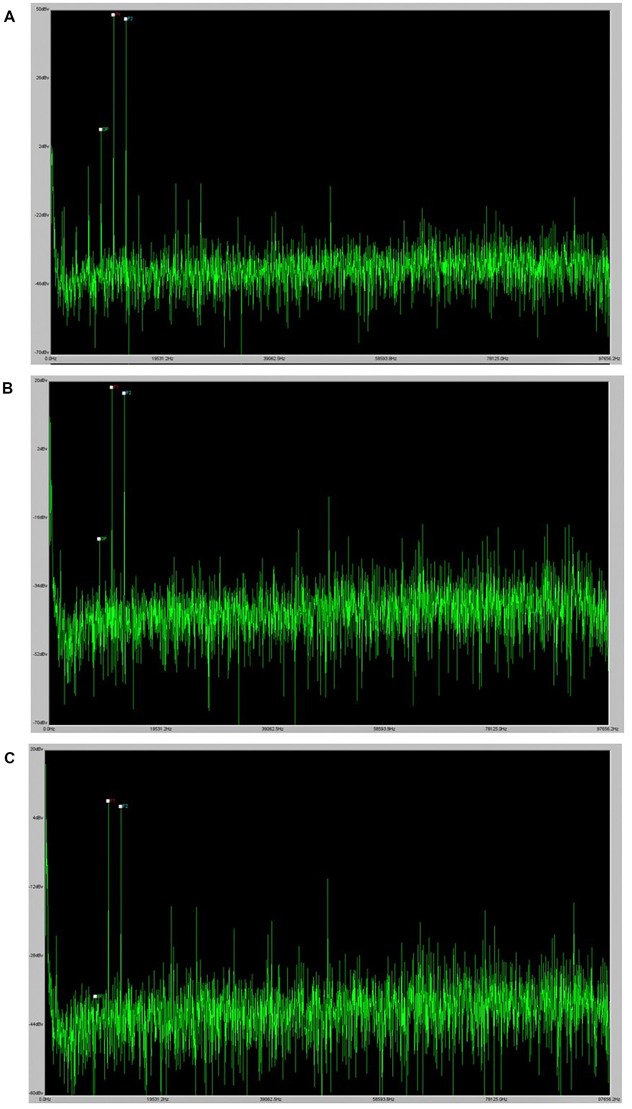
DPOAE example. Panel **(A)** provides an example of a robust DPOAE as it displays in the BioSig software. Panels **(B)** and **(C)** provide examples of the visual indication of a DPOAE at the threshold and the absence of a DPOAE at lower levels.

While still under the isoflurane anesthesia after the ABR recording, DPOAEs were measured. Prior to recording DPOAEs, all animals were visually inspected for signs of middle ear infection or cerumen buildup within the external auditory canal. DPOAE measurements were collected at the same f_2_ frequencies as for ABR (4, 8, 12, 16, 24, and 32 kHz) with a ratio of f_2_/f_1_ constant at 1.25 and a ratio of L1/L2 constant at 1.2. At each frequency, stimuli began at 80 dB SPL for L1 and decreased in 10-dB steps to 20 dB SPL or until no cubic DPOAE (2f_1_-f_2_) response was observed. A cubic DPOAE was considered to be present if there was a visible spike at 2f_1_-f_2_ that exceeded the noise floor at nearby frequencies, as can be seen in the example in Figure 1. The lowest intensity at which a visible cubic DPOAE could be detected was recorded at each tested frequency and was defined as the DPOAE threshold for that frequency. The stimuli were generated using TDT SigGen software. Signals were routed to two speakers (TDT Model MF1) in a closed field configuration that were coupled to the microphone tip of the Etymotic Research ER10B+ low noise microphone system (Elk Grove Village, IL) using 1/16” inner diameter, 1/8” outer diameter plastic tubing (McMaster-Carr, Cleveland, OH). The microphone tip was coupled to the ear of each mouse using a pipet tip that was trimmed to fit the ear. For each level, DPOAE recordings were averaged across 128 responses at each level as recommended by the software manufacturer (TDT). Gain for responses was set at 0.00001 so that plot outputs matched dBv for simple conversion to dB SPL and F1, F2, and DP (2f_1_-f_2_) were labeled for all collected responses.

For breeding pairs, ABRs and DPOAEs were recorded prior to assigning each mouse to an experimental group, and then 1, 3, and 6 months after pairing the mice and beginning HAART exposure. For offspring, ABRs and DPOAEs were recorded at 28 days post-birth. Day 28 was selected as the test date because the mice were weaned from their birth cages at 21 days, and then the additional week was given for them to acclimatize to their new cages before undergoing anesthetized auditory testing. All auditory testing was performed during the day (between 9 a.m. and 6 p.m. Eastern time).

### Statistical Analyses

For the breeder females’, a three-way analysis of variance (ANOVA) comparing group × frequency × test time (0, 3, or 6 months since enrollment) was used. Frequency and test time were treated as within-subjects factors, with the group as the between-subjects factor. ABR and DPOAE thresholds were analyzed for differences by exposure group at wean using a two-factor ANOVA (group*frequency). When significant effects were observed, all *post hoc* analyses for the group used Tukey A pairwise comparisons. Significance was assigned at *p* < 0.05 for all analyses. All statistical analyses were performed using IBM SPSS version 25 (IBM, Armonk, NY) and all associated figures were created using SigmaPlot (Systat Software Inc., San Jose, CA).

## Results

### Breeding Pairs

**Figure 2:**
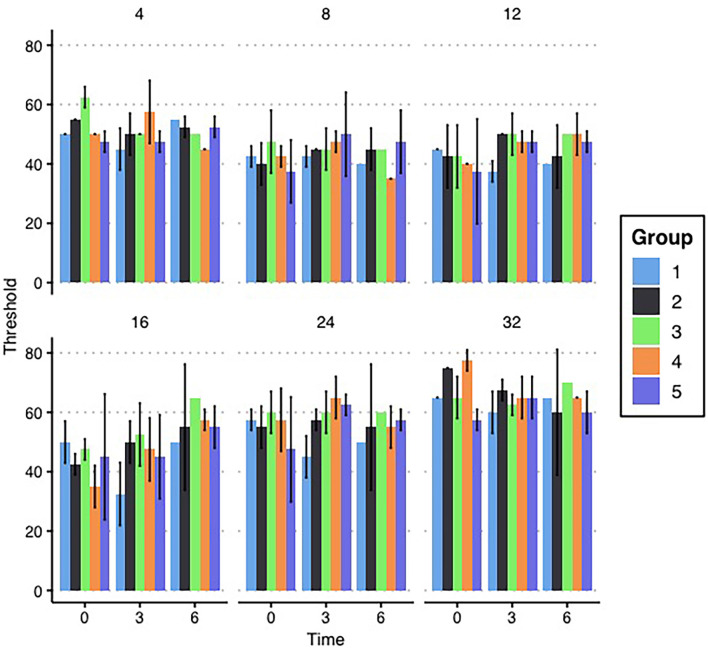
Breeder ABR results. Mean ABR thresholds for each group of female breeding mice are depicted by the bars in each panel. Thresholds are grouped with the first set in each panel depicting mean thresholds before enrollment in the study and the next sets indicating the ABR thresholds at 3 and 6 months, respectively. Each panel represents a tested frequency. No significant differences exist between groups. Error bars represent ±1 standard deviation (SD).

At the beginning of the study, the auditory status of all 20 breeding animals was evaluated *via* ABR. No animals were found to have abnormally high baseline ABR thresholds at enrollment, as can be seen in Figure 2. For the pre-exposure ABR thresholds, a two-factor ANOVA (frequency*ARV exposure group) was performed to determine differences in baseline hearing between groups’ breeder females. There was neither a two-way interaction of frequency and group (*F*_1,20_ = 1.106, *p* = 0.401) nor a main effect of group (*F*_1,4_ = 0.608, *p* = 0.675). This lack of differences between breeding mice across groups indicates that any differences seen in offspring are likely the result of ARV exposures and not the result of obvious inherent differences. While the female breeding mice were being exposed to daily antiretrovirals by gavage, their ABR thresholds were monitored throughout the duration of the study. ABRs were collected at 1, 3, and 6 months (see Figure 2 for means) after beginning the exposures in order to monitor any auditory changes resulting from HAART. A three-way repeated measures ANOVA (time*frequency*group) was performed to evaluate threshold changes across groups and frequencies over time in the HAART-exposed female breeding animals. There was no three-way interaction of group, frequency, and time (*F*_1,40_ = 0.846, *p* = 0.693) nor any two-way interaction of frequency and group (*F*_1,20_ = 0.662, *p* = 0.808). The only significant interaction was a two-way interaction of time and frequency (*F*_1,10_ = 2.734, *p* = 0.016). Evaluation of this effect showed a significant change in the mean threshold at 16 kHz between the 3-month and 6-month time points across groups. No other significant differences were observed, as can be seen in Figure 2. Overall, the results indicate that the daily ARV gavages did not create significant hearing threshold changes and that the mice exhibited generally stable thresholds, consistent with expectations for the CBA/CaJ mouse.

### Offspring

**Figure 3:**
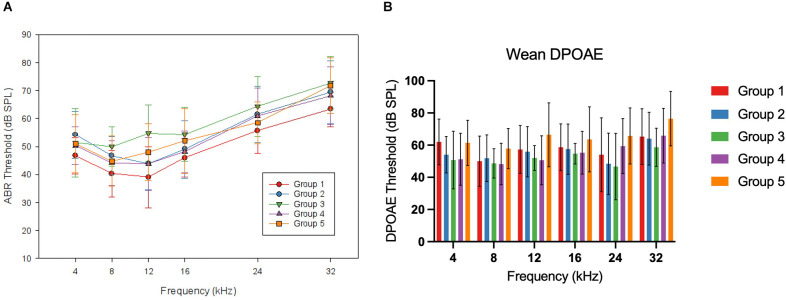
WeanABR and OAE. Panel **(A)** shows the mean ABR thresholds foreach group of offspring mice at wean. No frequency-specific differences were reported at this time point. Mean thresholds for Group 1 were lower than in Groups 2, 3, and 5 (*p* < 0.05). Panel **(B)** shows the mean DPOAE thresholds for each group of offspring mice at wean. No significant differences were seen at this time point. Error bars represent ±1 SD.

The target group size for each exposure group (Groups 1–5) was eight mice. Due to differences in litter sizes, this number served as a target, but the achieved group sizes for each exposure group varied slightly. ABRs and DPOAEs were measured 7 days after wean (28 days of age) for all offspring in the study. This timepoint is referred to as “wean” throughout the rest of this manuscript, and it reflects the auditory status as it was first measured after weaning these animals. Mean ABR and DPOAE thresholds at wean are depicted below in Figure 3, and group comparisons are described below.

For the ABR thresholds at this wean time point, a two-factor ANOVA (frequency*group) was performed. There was no two-way interaction (*F*_(1,17.685)_ = 1.222, *p* = 0.241). To account for a lack of sphericity, a Huynh-Feldt correction was run on the reported two-way interaction. A significant main effect was seen for the group (*F*_(1,4)_ = 4.749, *p* = 0.002). When evaluating the wean average threshold across frequencies by group with Tukey A *post hoc* comparison, the control group (Group 1) had lower thresholds than Group 2 (5.63 dB mean difference, *p* = 0.043), Group 3 (9.39 dB, *p* = 0.001), and Group 5 (5.83 dB, *p* = 0.049). DPOAE thresholds for this time point were analyzed using a two-factor ANOVA (frequency*group). There was no two-way interaction of group and frequency (*F*_(1,13.810)_ = 1.1622, *p* = 0.073) and there was no significant effect of group (*F*_(1,4)_ = 2.363, *p* = 0.059). To account for a lack of sphericity, a Huynh-Feldt correction was run on the reported two-way interaction with no change in the significance.

**Figure 4:**
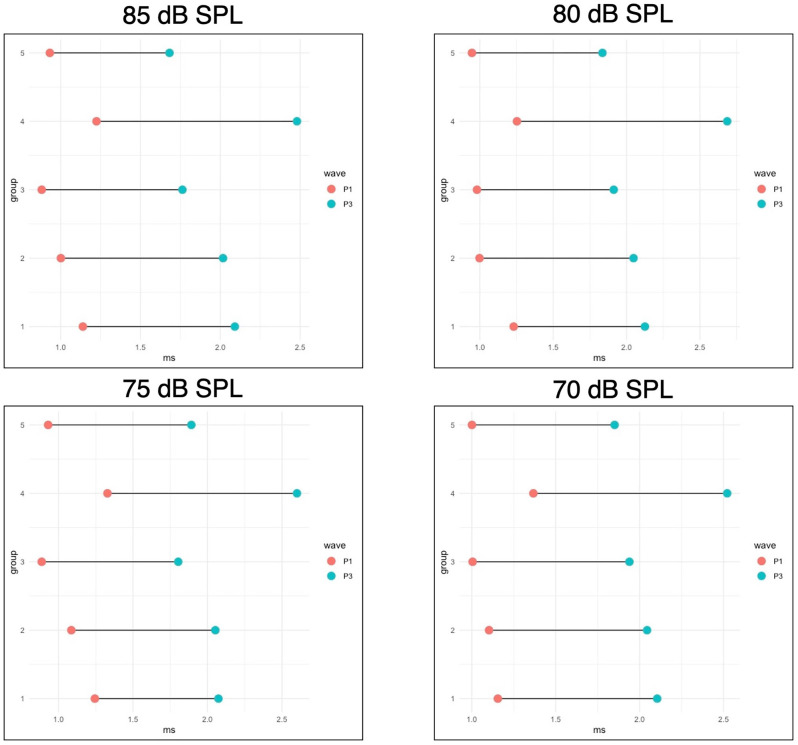
WeanABR latencies. This figure shows the mean P1 and P3 latencies foroffspring measured at wean for four intensities (90 dB SPL is notpictured, but no significant differences were seen at this level). P1-P3 interpeak latencies are represented by the bars connecting the points for each group. Group 4 had a greater P1 latency than Group 3 at 75 dB SPL, a greater P3 latency than groups 2, 3, and 5 at 75–85 dB SPL and than Group 1 at 75–80 dB SPL, and a greater P1-P3 interpeak latency than Group 1 at 75 dB SPL, Groups 1, 2, 3, and 5 at 80 dB SPL, and than Groups 3 and 5 at 85 dB SPL. All significant differences are *p* < 0.05.

To physiologically evaluate the consequences of HAART exposure during PaB on the afferent synaptic pathway and auditory brainstem, P1 and P3 latencies, and P1-P3 interpeak latencies were evaluated for all responses from 70 to 90 dB SPL at 16 kHz for all animals at wean. 16 kHz was chosen for this measure because it had robust responses and low thresholds in all experimental groups, and so was considered likely to indicate if there were any suprathreshold effects across groups. A one-way ANOVA (group) found a significant effect of group for P1 latency at 75 dB SPL (*p* = 0.036) and for P3 latency and P1-P3 interpeak latency at 75 (*p* = < 0.001, 0.021), 80 (*p* = 0.001, < 0.001), and 85 dB SPL (*p* = 0.001, .011). When evaluating these differences using Tukey A *post hoc* analysis, Group 4 had a greater P1 latency than Group 3 at 75 dB SPL, a greater P3 latency than Groups 2, 3, and 5 at 75–85 dB SPL and than Group 1 at 75–80 dB SPL, and a greater P1-P3 interpeak latency than Group 1 at 75 dB SPL, Groups 1, 2, 3, and 5 at 80 dB SPL, and than Groups 3 and 5 at 85 dB SPL. These results can be seen in greater detail in Figure 4.

## Discussion

### Exposure to AZT and EFV During PaB Leads to Elevated ABR Thresholds at Wean

This study represents the first evaluation of the risk associated with exposure to specific controlled antiretrovirals during PaB. The authors hypothesized that antiretroviral exposure during PaB would cause elevated ABR thresholds, with the greatest elevation arising from exposure to AZT and EFV. The results indicate that this hypothesis was correct, as both groups receiving either AZT or EFV had higher thresholds than controls. The group receiving both EFV and AZT saw an additional roughly 4-dB threshold mean increase over the groups receiving only one of those drugs. It is important to acknowledge that while Group 3 (AZT+EFV+3TC) saw higher thresholds than any other group in the study, those thresholds did not reach the level of statistical significance when compared to other ARV-exposed groups. While Group 4 (TDF+NVP+3TC) did not see elevated thresholds when compared to the control group, this group saw increased P3 and interpeak latencies when compared to all other groups. This indicates that different antiretroviral combinations may have different auditory impacts related to exposure during pregnancy and breastfeeding, and the differences across auditory measures may help to distinguish the site of lesion for these exposures.

The hypotheses of this study were largely driven by findings of toxicity to auditory cell lines *in vitro* (Thein et al., 2014). In that study, the authors investigated the mechanisms driving observed ototoxicity and were able to state that auditory cell losses seemed to be caspase 3/7-independent, indicating that those pro-apoptotic pathways did not appear to be the drivers of cell death. They hypothesized that, since EFV did not bind to mitochondrial DNA polymerase-y, EFV-induced damage was likely the result of endoplasmic reticulum stress. Subsequent exploration of the mechanisms of EFV-driven cytotoxicity has found that EFV causes significant cellular instability through the permeabilization of the mitochondrial outer membrane and induces changes in the mitochondrial membrane potential (Ganta et al., 2017). Changes in the mitochondrial membrane potential lead to cytochrome c release and mitochondrial-mediated apoptosis, both known causes of outer hair cell loss (Wang et al., 2004). ARV exposures have also shown toxicity to the placenta (Collier et al., 2003) and other organs throughout the body (Benbrik et al., 1997), which may lead to auditory impacts as a result of this damage. While the current study did not directly evaluate the cellular mechanisms driving threshold elevation, these results when combined with the current literature, suggest that one or more mechanisms may be synergistically combining to damage the auditory system of those exposed to these drugs during PaB.

The lack of significant differences in DPOAE thresholds for this study indicates that the ABR threshold elevations were not the result of damage to the OHCs and were instead the result of damage to the inner hair cells, auditory nerve, and/or auditory brainstem. This is in line with previous work on the auditory impacts of ARVs that found abnormalities in the morphology of the ABR thought to be indicative of central auditory system pathology (Matas et al., 2010) and our previous experiment in the C57Bl6/J mouse (DeBacker et al., 2022). While this contradicts the findings of Thein et al. (2014), it is possible that differences in route of administration, cochlear supporting structures, or *in utero* delivery vs. direct administration to *ex vivo* samples may have caused these differences. This is further supported by the differences in P3 latency and P1-P3 interpeak latency seen in Group 4 when compared to other exposure groups. Especially since Group 4 had no significant differences in ABR threshold at wean, these significant differences in ABR morphology indicate that antiretroviral exposure during PaB may be causing auditory dysfunction that is not detected using conventional hearing screening methods.

Likely the most significant implication of this work is that ARV exposure during PaB causes auditory dysfunction that would not be detected in the most common newborn hearing screenings with ABRs or DPOAEs. This may help to clarify the currently mixed findings in the literature for PHEU children. The Torre group found impaired auditory processing in young adults who had been exposed to HIV and HAART *in utero* (Torre et al., 2020), but did not find an increased rate of failed hearing screenings in children exposed to HIV and HAART *in utero* (Torre et al., 2017). These findings could indicate that children exposed to HAART *in utero* are not at increased risk for clinically-significant hearing loss, but are at risk for other auditory processing difficulties. It is important to note, however, that hearing screening at birth is not as sensitive as other auditory measures, like diagnostic threshold ABRs, and so is likely to miss subtle differences resulting from *in utero* HAART exposure. The small threshold elevations seen in the mice in this study would be unlikely to cause a failed hearing screening, especially given the lack of impact on DPOAEs, which are frequently used in newborn hearing screenings. As such, it is possible that if such minimal hearing losses are also occurring in PHEU children, they are being missed on early hearing evaluations. PHEU children are therefore unable to benefit from the early intervention they would have received had these hearing differences been caught at birth. There is evidence linking short-term minimal hearing losses from otitis media to long-term auditory processing difficulties (Moore et al., 2020), and so it is possible that persistent minimal hearing losses like those seen in this study could result in the auditory processing difficulties described by Torre et al. (2020).

### No Threshold Shifts Were Seen in Adult Mice

No auditory differences were seen in adult mice as a result of ARV exposure during the study. This is in agreement with previous studies of ARV exposure in mice (Bektas et al., 2008), and it is consistent with a model that indicates ARV exposure is causing subtle, even sub-clinical, changes in hearing during development. The CBA/CaJ mouse has “golden ears,” a term used to indicate that they develop little age-related hearing loss. Certainly, within the time window of the breeding for the experiments, the female breeder mice would not have been expected to develop age-related hearing loss, and indeed they did not. Our previous work in this area used the C57Bl6/J mouse (DeBacker et al., 2022), which develops age-related hearing loss within weeks of its wean age (Willott et al., 1991). The variability in thresholds of those mice makes it difficult to interpret small mean differences of less than 10 dB. However, the same differences were detected in the CBA/CaJ mouse, which significantly reinforces the earlier finding. There was no reason to expect the HAART-exposed CBA/CaJ mice to be different from the control group unless the HAART exposure during PaB was indeed affecting the auditory system.

### Limitations

It is of course important to recognize that the current study was a pre-clinical model of hearing using the CBA/CaJ mouse. While this is a well-studied model of audition and ototoxicity and every effort was made to design this study to be translational in nature, there are limitations inherent to non-human animal studies when applying the results to human populations. As such, further study in humans is required to confirm these findings are applicable across species. Additionally, it should be recognized that this was a model of HAART exposure only and not of the combined effects of HIV and HAART on the developing offspring. As such, differences may exist when introducing the variable of HIV infection alongside these exposures, and future studies should evaluate these exposures concurrently to determine if the addition of HIV impacts the auditory effects seen in this study. Lastly, it should be recognized that the mean threshold elevations seen in this study are small (5–9 dB SPL). Despite the small degree of hearing impairment, the literature on language and cognitive development in children discussed above indicates that even these minimal hearing losses can have significant impacts on outcomes in children. This impact on outcomes is particularly concerning given the fact that minimal hearing losses like those observed in this study are less likely to be detected, even in settings with robust early hearing screening protocols.

## Conclusions

The current study found that exposure to HAART, especially cocktails including AZT and EFV, during PaB was associated with increased ABR thresholds and differences in ABR wave latencies at wean when compared to unexposed offspring. These same threshold elevations were not seen on DPOAEs. Due to the minimal degree of threshold elevation (5–9 dB SPL) and lack of impact on DPOAEs, these hearing losses would be unlikely to be detected with common newborn hearing screenings. This may explain some of the discrepancies in the current literature relating to auditory function at birth in PHEU children (Poblano et al., 2004; Fasunla et al., 2014; Torre et al., 2017). Further study in models of concurrent HIV and HAART exposure and in human subjects is warranted to confirm the clinical relevance of these results.

## Data Availability Statement

The raw data supporting the conclusions of this article will be made available by the authors, without undue reservation.

## Ethics Statement

The animal study was reviewed and approved by Ohio State University Institutional Animal Care and Use Committee.

## Author Contributions

JD and EB contributed to the conception and design of this study. JD and BL performed experiments and collected data. JD performed statistical analyses and wrote the first draft of the manuscript. BL and EB wrote sections of the manuscript. All authors contributed to the article and approved the submitted version.

## Conflict of Interest

The authors declare that the research was conducted in the absence of any commercial or financial relationships that could be construed as a potential conflict of interest.

## Publisher’s Note

All claims expressed in this article are solely those of the authors and do not necessarily represent those of their affiliated organizations, or those of the publisher, the editors and the reviewers. Any product that may be evaluated in this article, or claim that may be made by its manufacturer, is not guaranteed or endorsed by the publisher.

## Abbreviations

3TC: lamivudine
ABR: auditory brainstem response
AIDS: acquired immunodeficiency syndrome
ANOVA: analysis of variance
AZT: zidovudine
ARV: antiretroviral
DPOAE: distortion product ototacoustic emissions
EFV: efavirenz
HAART: highly-active antiretroviral therapy
HIV: human immunodeficiency virus
NRTI: nucleoside reverse transcriptase inhibitor
NVP: nevirapine
OHC: outer hair cell
PaB: pregnancy and breastfeeding
TDF: tenofovir disoproxil fumarate
TDT: Tucker Davis Technologies
WHO: World Health Organization.
